# Supplementary material for: Chromothripsis during telomere crisis is independent of NHEJ, and consistent with a replicative origin
Source: Genome Res. 2019 May;29(5):737–49. doi: 10.1101/gr.240705.118 (PMC6499312; doi:10.1101/gr.240705.118)
Supplement: Supplemental Material [file supp_gr.240705.118_Supplemental_file_1.zip › contigs/annotated_contigs/DB51/contig.2.DB51_length_538_mean_cov_4.61710037175.docx]

**DB51_length_538_mean_cov_4.61710037175**

```
TTTGTATACTAAAGAGTTTAGATTTATTCTGGATAAATGGAGAGCTGTGGAAGGATTTTAAAGGGAAGAGTGATTTAAAAAGACAATAA
 >chr3:180299102-180299278 - E=3e-91
CGTGGCTAATAGAACATTTTAAATTACATATGTGTTCACGTTATATTTCTATTGGCCAGTTTCTATTTGGATATCCTGTCATCAGTC|C

TTATATAGTCATGGTCAGAGATGAAATTCTTTATTTGGAGGTAAAGAGTTAAATGTAGACATATAGGCAGGAAAAAAAAAACCAGCATG
 >chr3:180297656-180298018 - E=3e-200
AAAGCACACTATGTACATTAGAAGAGTTGAAGAGAAATGAGCCTTATCATGCAAACCACATTGACTTTGAAACATATTGCAGTCATATT

GTTCTGAAATTCTTATTGTAGGATACATATTGAGATTGTCAGAAAGGCTGTGTGAACACTGGGGCTTTTGCAGCACTGACACTATTTTT

ACCTATTTTGTTGCTTTGGCTTTTGAGCTCTCTTCCCTCCTTTCTTCCTTCCTTCCTTCCTTCCTTTCTTTCTTTCTCTCTCTTTCTGT

CTTTC
```
